# Novel Surgical Technique for Total Knee Arthroplasty Integrating Kinematic Alignment and Real-Time Elongation of the Ligaments Using the NextAR System

**DOI:** 10.3390/jpm14080794

**Published:** 2024-07-26

**Authors:** Luigi Sabatini, Daniele Ascani, Daniele Vezza, Alessandro Massè, Giorgio Cacciola

**Affiliations:** 1 Robotic and Minimally Invasive Orthopaedic Center, Humanitas “Gradenigo”, 10153 Turin, Italy; luigisabatini.ort@gmail.com (L.S.); daniele.vezza@unito.it (D.V.); 2 Medacta International, 6874 Castel San Pietro, Switzerland; ascani@medacta.ch; 3 Department of Orthopaedic and Traumatology, University of Turin, 10125 Turin, Italy; alessandro.masse@unito.it

**Keywords:** kinematic alignment, augmented reality, NextAR, personalized arthroplasty, enabling technology

## Abstract

This study introduces an innovative surgical approach for total knee arthroplasty (TKA) that combines kinematic alignment (KA) principles with real-time elongation of the knee ligaments through the range of motion, using augmented reality (AR). The novelty of the surgical technique lies in the possibility of enhancing the decision-making process to perform the cut on the tibia as for the KA caliper technique developed by Dr. Stephen Howell. The NextAR is a CT-based AR system that offers the possibility of performing three-dimensional surgical preoperative planning and an accurate execution in the surgical room through single-use infrared sensors, smart glasses, and a control unit. During the preoperative planning, the soft tissue is not considered and only the alignment based on bony reference is ensured. Thanks to the possibility of measuring in real time the elongation of the knee collateral lateral ligaments, the system assists the surgeon in optimizing the cut on the tibia after an accurate resurfacing of the femur as described in the KA surgical technique. The implant used in this novel approach is a medial pivot design (Medacta GMK Sphere) that allows the restoration of the physiological behavior of the software tissue and natural knee kinematics. In conclusion, this novel technique offers a promising approach to TKA, allowing personalized treatment tailored to each patient’s unique anatomy and soft tissue characteristics. The integration of KA and real-time soft tissue analysis provided by NextAR enhances surgical precision and outcomes, potentially improving patient satisfaction and functional results.

## 1. Introduction

In this paper, we present a surgical technique for total knee arthroplasty (TKA) that blends the principles of kinematic alignment (KA) and the use of an augmented reality (AR) device that provides the real-time elongation of the collateral lateral ligaments of the knee (NextAR, by Medacta International, Castel San Pietro, Switzerland). KA is a “personalized alignment” that aims to restore as close as possible to the pre-arthritic joint line of each patient [1,2,3,4]. Unlike conventional mechanical alignment techniques, which aim to align the knee joint to a universally neutral axis, KA tailors the positioning of the knee replacement components to fit the patient’s specific anatomical structure to achieve a more natural postoperative kinematic [1,2,3,4]. While resurfacing the femur during knee replacement surgery typically allows for predictable restoration of the femoral joint line due to the predictable nature of femoral wear, this is not always straightforward for the tibia [5,6,7]. The tibial joint line can be more challenging to assess and restore accurately because tibial wear patterns are less consistent and more variable [5,6,7,8].

**Figure 1:**
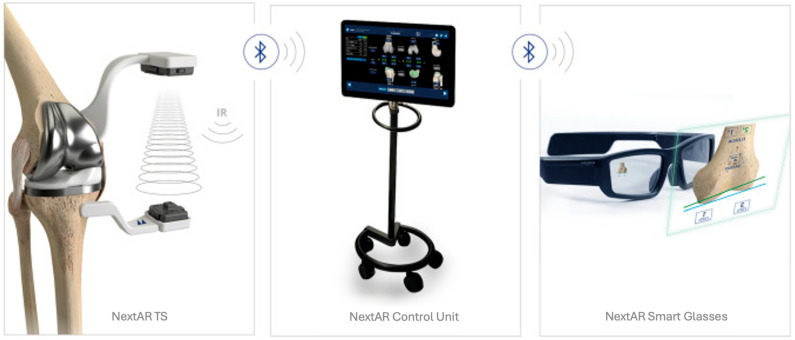
The NextAR System (Medacta International).

NextAR is a CT-based AR navigation system, that consists of a pair of smart glasses, two small single-use infrared sensors, and a control unit (Figure 1) [9]. The use of the head-mounted display allows the superimposition on the surgical field of relevant surgical information, such as the position of the cutting guides and thickness of the bone cuts while surgical procedures are performed. One of the main advantages of this system is the possibility to evaluate intraoperatively the elongation of the medial (MCL) and lateral collateral ligament (LCL) [10,11]. Currently, no studies have been published using the NextAR in TKA. However, in recent years, several authors have begun exploring the use of AR in TKA across both preclinical and clinical settings [5,12,13,14,15].

The MCL is a primary stabilizer of the knee, responsible for resisting valgus stress. Many kinematic studies, made with a different technique in both cadaver specimens and in vivo, demonstrate that during knee flexion the MCL fibers undergo minimal length changes. The different bundles of the MCL become tighter or lax at varying degrees of knee flexion but functionally maintain knee stability against valgus stress during the whole range of motion [16,17,18]. On the other hand, the LCL that is tightened in full extension, resisting varus stress, became a bit more lax in deep flexion (corresponding to a shortening of the ligament) [19,20]. The combination of elongation and strain stress resistance of the MCL and LCL makes the knee stable in both flexion and extension, allowing for a minimal laxity on the lateral side of the knee that is responsible for the medial pivot kinematics of the native knee [21,22].

This technique was proposed as a computer-assisted surgical procedure to the unrestricted KA proposed by Howell [23]. The concept of soft-tissue elongation tibial cut aims to restore the preoperative distal and femoral joint line and to restore the native MCL and LCL elongation during the whole range of motion. This technique is based on three principles. The first principle is to fully resurface the femur (same as unrestricted KA described by Howell [23]. Resurfacing the distal and posterior femur results in removing the same amount of bone and cartilage of the thickness of the implant (matching the thickness in non-worn compartments and compensating for loss in worn compartments) allowing the restoration of the three kinematic axes of the femur (cylindrical or transcondylar axis and the patellar flexion axis). The second principle is to analyze the MCL and LCL elongation after performing femoral resurfacing with femoral trial components in situ. After positioning the trial component and performing femoral resurfacing, we restored the distal and posterior joint line of the femur. Therefore, by evaluating the length of the collateral ligaments throughout the entire range of motion, we can obtain important information on how to adjust the tibial cut planning. This ensures a stable knee under varus/valgus stress in extension (with less than 1 mm opening both medially and laterally), while also allowing for medial pivot in flexion. The third principle is to restore a more native knee kinematic as possible by the use of a medial pivot liner design. In this study, we used a femoral component designed specifically to optimize femoral coverage and patellar tracking in KA (GMK SpheriKA, by Medacta International, Castel San Pietro, Switzerland) and a spherical liner characterized by a medial ball-in-socket stability thanks to 1:1 congruency with the femoral condyle and an unconstrained lateral compartment designed to permit complete freedom of rollback and rotation as knee flexes.

## 2. Surgical Technique

### 2.1. Case Presentation

We present the case of a 67-year-old woman diagnosed with primary knee osteoarthritis requiring TKA. Before the procedure, she underwent a preoperative CT scan of the pelvis, knee, and ankle. Her limb alignment was classified as CPAK II (neutral apex distal), with a hip-knee angle (HKA) of 173.7°, a lateral distal femoral angle (LDFA) of 87.6°, a medial proximal tibial angle (MPTA) of 86.6°, an arithmetic HKA (aHKA) of −1°, a joint line obliquity (JLO) of 174°, and a posterior tibial slope (PTS) of 8.8°. The patient received spinal anesthesia. For infection prevention, a prophylactic dose of 2 g of cephazolin was administered preoperatively. Additionally, to control bleeding, 1 g of tranexamic acid was given at the time of the incision, with an additional gram administered 6 h post-surgery to reduce blood loss.

### 2.2. Three-Dimensional Preoperative Planning

**Figure 2:**
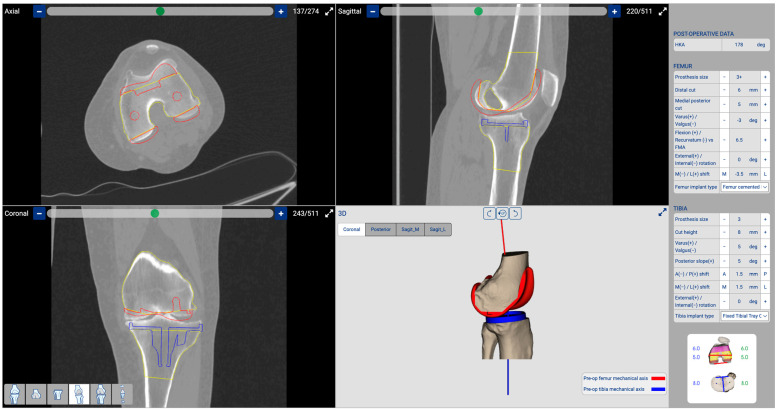
Three-dimensional planning: The software enables us to conduct three-dimensional planning based on a CT scan. To preserve the native LDFA, we planned a distal femoral cut at 2.5° valgus with a bone thickness of 6 mm. The posterior femoral cut was set at 0° of rotation with a bone thickness of 5 mm. The tibial cut was planned with a varus of 3.5° and a PTS of 8.5°. Additionally, we determined the optimal component sizes, selecting a femoral component size 3+ and a tibial component size 3. In red is represented the position of the planned femoral component, while in blue is the position of the tibial component.

Based on Howell’s unrestricted KA criteria, we devised a strategy for femoral resurfacing by removing an equivalent amount of bone and cartilage to match the thickness of the prosthesis. Our plan involved a pure resurfacing of the femoral component on the coronal plane while maintaining the native LDFA. Additionally, we set the posterior femoral cut on the axial plane at 0° rotation from the posterior condylar axis. In the sagittal plane, we adjusted the flexion/extension to the optimal position to prevent femoral notching. In a varus knee, as described in the case reported in this technical note, the wear is typically 2 mm of cartilage on the medial femoral condyle [24], while there is no cartilage wear on the lateral femoral condyle. To match the thickness of the prosthesis, we determined that 6 mm of bone needs to be removed from the medial femoral condyle (6 mm of bone + 1 mm saw thickness + 2 mm of missing cartilage equals the 9 mm thickness of the distal prosthetic medial femoral condyle). For the lateral femoral condyle, we remove 8 mm of bone and cartilage (6 mm bone + 2 mm cartilage + 1 mm saw thickness equals the 9 mm thickness of the distal prosthetic lateral femoral condyle). For the posterior condyle, we remove 2 mm less than the thickness of the prosthetic posterior femoral condyles (3 mm bone + 2 mm cartilage + 1 mm of saw thickness, which is 2 mm less than the 8 mm thickness of the prosthetic posterior femoral condyles). Increasing the posterior condylar offset by 2 mm is necessary when using a liner that involves the removal of the posterior cruciate ligament (as the one used in this case). This adjustment helps close the flexion gap and prevents a mismatch between extension and flexion. Regarding the tibia, our initial plan involved pure resurfacing, and maintaining the native MPTA and the native posterior slope. However, the positioning of the tibial cut would be adjusted or confirmed based on soft tissue elongation information acquired during the surgical procedure following femoral resurfacing with trial component placement (Figure 2).

### 2.3. NextAR TKA System

The NextAR system has a compact design, consisting of augmented reality smart glasses, two small single-use sensors, and a control unit (Figure 1). The smart glasses allow for the display of the field of view real-time information compared to the preoperatively planned values needed to perform the surgical steps. The augmented reality feature improves the user experience by avoiding the continuous look at the external monitor and favoring hand-eye coordination. The smart glasses are lightweight, fully wireless, and equipped with an integrated battery. The information is displayed in a straightforward interface that does not cause fatigue or headaches even after prolonged use [10,11].

The surgical guidance is performed by a wireless optical tracking system that comprises two small-sized sensors: an active infrared camera and an active tracker that eliminates the need for external cameras and any line-of-sight issues. The two sensors are provided in a sterile, single-use format and are ready to be used without the need for calibration, the battery life is certified for over 4 h. When the tracker is correctly positioned, it transmits spatial data in six degrees of freedom (three translations, three rotations) with an error ≤0.5°/0.5 mm, the software warns the user when the tracker approaches the boundaries of the recommended volume of measurement to maintain maximum accuracy [10,11]. The camera and tracker can be securely attached to the femur and tibia within the surgical incision, eliminating the need for percutaneous bone pins, throughout the procedure, they provide guidance to the surgeon via the smart glasses.

Completing the hardware of the NextAR TKA is a control unit connected via Bluetooth to the tracking systems and smart glasses. Once the initial setup is complete, the control unit is only necessary if the surgeon decides to modify the surgical plan, as all other steps can be monitored and visualized by utilizing only the sensors and smart glasses

### 2.4. Five Steps Technique

**Figure 3:**
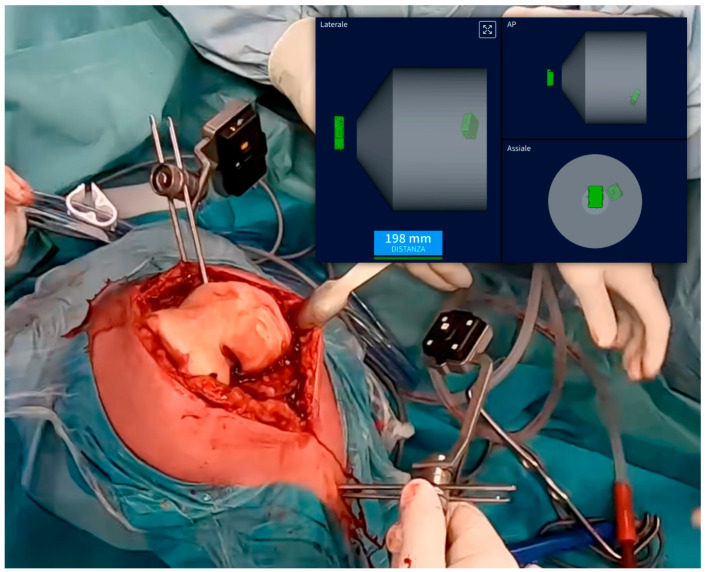
Camera holder positions: The tibial camera holder is positioned 8 cm below the joint line perpendicular to the joint line, while the femoral camera holder is situated approximately 5 cm above the femoral joint line at a 45° angle; in the image, the top right displays real-time data ensuring that the two cameras, integral to the AR system, maintain contact (in green). It is mandatory to verify that these cameras remain in contact throughout the entire range of motion.

**Figure 4:**
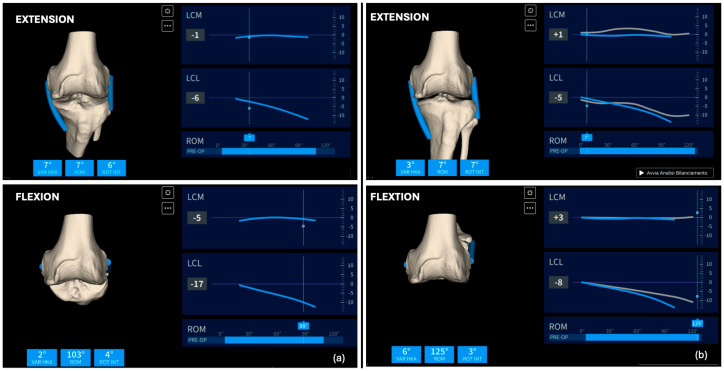
(**a**) (**top**) Collateral ligament elongation in extension (**top**) and in flexion (**bottom**) showing a shortened MCL before the femoral resurfacing. (**b**) Collateral ligament elongation after femoral resurfacing shows the restoration of more natural kinematics in extension (**top**), flexion (**bottom**), and during the whole range of motion.

Surgical Approach and Device Registration (Step 1): The surgical approach involves a standard median skin incision and a medial parapatellar arthrotomy. During this approach, care must be taken not to remove the femoral and tibial osteophytes, as preserving the bone surface is crucial for accurate registration with the preoperative CT scan. After adequately exposing the joint, position the femoral and tibial pins. The femoral pins should be placed approximately 5–6 cm from the joint plane, oriented at about 45° to the ground, while the tibial pins should be positioned outside the surgical field, oriented perpendicularly and approximately 8 cm from the tibial plateau (Figure 3). Camera holders, which will house the single-use sensors, are then placed on the pins. Before proceeding with the bone surface registration, ensure that the distance and orientation between the two sensors are adequate throughout the entire range of motion. The system employs a single-point acquisition method, wherein a pointer is held on the relevant anatomical structure to activate automatic point acquisition. Twenty-six points each for the femur and tibia need to be registered to align the preoperative CT scan with the intraoperative bone surface. After completing bone registration, the femoral and tibial osteophytes may be removed before assessing soft tissue elongation. To establish the reference length (L0) of the collateral ligaments, the knee should be held between 0° and 10° of flexion. Once L0 is determined, ligament length variation can be evaluated throughout the range of motion (ROM). Applying varus/valgus stress across the entire ROM helps define the limits of maximum elongation and shortening of the collateral ligaments (boundaries). In a varus knee characterized by medial cartilage wear of the distal condyle, it is expected that the medial collateral ligament (MCL) will not be tight in extension due to the cartilage wear (Figure 4a).

**Figure 5:**
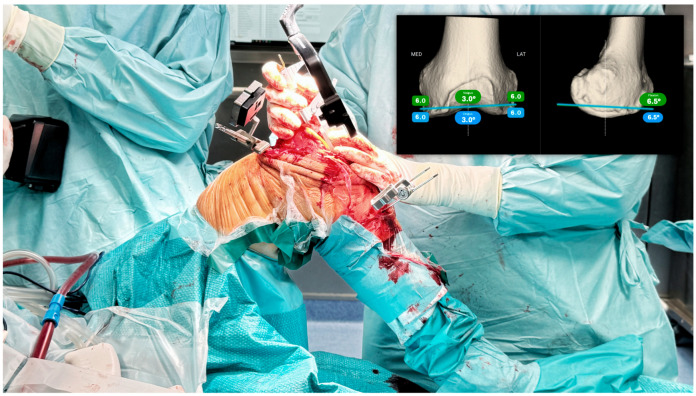
Navigation of the distal femoral cut involves aligning the guide with the planned position (indicated by a green line) using real-time position information (indicated by a blue line) in both the coronal and sagittal planes.

Femoral Resurfacing (Step 2): This procedure involves performing femoral resurfacing as described in Howell’s KA unrestricted technique. The only difference from the traditional technique is the necessity to increase the posterior condylar offset by 2 mm (removing 2 mm of bone less than the thickness of the prosthesis) if one decides to sacrifice the posterior cruciate ligament (PCL). If the surgeon chooses to maintain the PCL removal of the same amount of bone as the thickness of the prosthesis is suggested. The “tibial” camera will be positioned on the MIKA distal cutting guide using the correct plate based on the cartilage wear (in this case medial wear and lateral underwear to make a 6 mm cut on the medial femoral condyle and 8 mm on the lateral femoral condyle). Using the AR system, we will navigate the position of the cutting guide, first matching the sagittal plane by adjusting the flexion/extension of the component, and then the varus/valgus alignment. Using the augmented reality system, we will navigate the position of the cutting guide, first matching the sagittal plane by adjusting the flexion/extension of the component, and then the varus/valgus alignment. Once the blue line, representing the real-time position of the guide in space, is parallel to the green line, representing the planned position of the distal cutting guide (Figure 5), we will stabilize the cutting guide with three pins (at least one of which is oblique) and proceed with the distal cut. Next, the posterior cut will be performed at 0° relative to the posterior condylar axis and 2 mm less than the thickness of the prosthesis to close the flexion gap for the reasons previously explained. This cut can be made manually using the MIKA instrument sizer or it can be navigated like the previous cut. Once the pre-planned four-in-one cutting guide is positioned, we will proceed with the cut. At this point, having completed the cuts for pure femoral resurfacing, the trial femoral component will be placed. At this stage, osteophytes can be removed before proceeding to the next step.

Definition of the tibial cut by having real-time soft tissue elongation (Step 3): With the trial component in place after performing the femoral resurfacing cuts (considering that femoral wear is predictable), we will evaluate the knee’s stability in extension and flexion, as well as the elongation of the medial and lateral collateral ligaments (Figure 4). Based on the intraoperative information acquired after femoral resurfacing, we will modify the planning of the tibial cut to achieve isometry of the MCL throughout the entire range of motion, and an LCL that matches the L0 length in extension and tends to shorten with increased flexion. This pattern of collateral ligament elongation corresponds to a clinical scenario where, in extension, there will be negligible opening (<1 mm) in both the medial and lateral compartments, while in flexion, the medial compartment remains stable (maximum valgus stress opening of 1 mm) and the lateral compartment may open up to 2–3 mm when a varus stress is applied, facilitating the natural medial pivot kinematics of the knee. We will adjust the tibial cut orientation on the coronal plane (adjusting varus/valgus), the sagittal plane (adjusting the slope), and the thickness of the tibial cut to achieve the desired elongation pattern. In this case, we modified the tibial varus from 1.5° to 2.5° and reduced the slope from 8.8° to 5°. Using the same navigation rules as the previous step, we will position the femoral camera on the MIKA extramedullary guide, correcting the position to match the tibial slope, varus/valgus, and finally the thickness. We will stabilize the guide with three pins (at least one oblique) and proceed with the tibial cut.

Evaluation of Soft Tissue Balance with Spacer Blocks and Trial Components (Step 4): As in the standard calibrated technique, we will place spacer blocks in flexion and extension to confirm the accuracy of the cuts. First, we will evaluate the flexion space with a 10 mm spacer, expecting to achieve a “medial pivot” where the spacer block pivots medially and moves more freely laterally. Next, we will place the 12 mm spacer block in the extension and verify that there is less than 1 mm of laxity under varus and valgus stress. We use the 12 mm spacer in extension instead of the 10 mm spacer because, at this stage, the posterior condyles are absent, reducing their tension on the posterior capsule. If satisfied with the balance using the spacer blocks, we proceed with preparing the tibial plateau. If not satisfied, we may perform additional recuts (only for the tibia). The same kinematic evaluations (stability in flexion/extension and range of motion) will be repeated with the trial components before proceeding to the definitive components implantation.

Components implantation and final evaluation (Step 5): Firstly, the tibial component (in this case, GMK Primary 3) will be implanted using a double cementation technique, where both the tibial plate and the lower portion of the tibial component are cemented with low-viscosity cement. Next, the final liner (in this case, the FLEX, 10 mm thick) will be positioned. Lastly, the femoral component (GMK SpheriKA 3+) will be cemented. A final assessment of the soft tissue elongation will be performed using the NextAR system. At the end of the procedure, the system will generate a detailed report of the planned and executed cuts and the elongation of the ligaments during the various phases of the surgery.

### 2.5. Postoperative Clinical and Radiographic Outcome

One year after the surgery, the patient is satisfied with the surgical procedure, the knee had a complete ROM (0°–135°) and is stable in flexion, mid-flexion, and extension. The Forgotten Joint Score Improved from 30 preoperatively to 81.7 postoperatively, while the WOMAC Score improved from 48 preoperatively to 13 postoperatively. Postoperatively her limb alignment was classified CPAK II (neutral apex distal, same as preoperative). The HKA changed from 173.7° to 178.5°, the postoperative LDFA was 87.5°, the postoperative MPTA was 86.5°, while the posterior PTS was 5.5°.

## 3. Discussion

This technique emphasizes three key principles: first, meticulous preoperative planning based on three-dimensional CT scans, focusing on bone rather than cartilage thickness for accurate cuts; second, a sequential approach involving femoral resurfacing followed by a collateral ligament’s elongation based tibial cut based on soft tissue information; and third, the use of a spherical liner.

The theoretical advantage of this technique is to restore both the native joint line orientation and the preoperative soft tissue tensioning, thereby reproducing more natural knee kinematics. Firstly, the CT scan of the pelvis, knee, and ankle provides detailed information about lower limb alignment, significantly enhancing preoperative planning. This allows for more precise planning and positioning of components using 3D planning instead of traditional 2D methods [25,26]. Secondly, the ability to intraoperatively verify the accuracy of the planned cuts can reduce the risk of errors at each step of the procedure [5,8]. Thirdly, obtaining real-time information about collateral ligaments, which is crucial in our technique for adjusting the tibial cut, can also be beneficial if the surgeon opts for either mechanical alignment or restricted kinematic alignment other than the above-mentioned technique [9].

In recent years, despite there being currently no studies about the accuracy of the NextAR device, interesting results in terms of accuracy were reported with the assistance of similar AR devices in TKA. Castellarin et al. reported an accuracy of the tibial cut of 0.59° ± 0.55° in the coronal plane and of 0.70° ± 0.75° on the sagittal plane with a different AR System (Knee+ Device) in a series of 76 patients (tibial cut first with the aim of maintaining the preoperative joint line) [27]. With the same device, Iacono et al. reported that the average error of their tibial cut was inferior to 1° on the coronal plane in 97% of cases and 88% in the sagittal plane [28]. Similar results were also achieved when AR devices were used to assist the femoral cuts. Tsukada et al. reported that the Knee+ AR device was non-inferior in accuracy compared with an accelerometer-based navigation system (109 knees versus 119 knees), reporting an accuracy of 95.4° in the AR group and of 93.2% of the navigation aiming for accuracy within 3° from the mechanical axis on the coronal plane [29].

By combining the precision of an AR system, with the possibility to have real-time information about soft tissue elongation. By achieving a balance between soft tissue tension and bony alignment, our goal is to restore pre-arthritic knee function while ensuring optimal stability and range of motion. Integrating KA and enabling technology enables individualized treatment tailored to each patient’s unique anatomy and soft tissue characteristics [16,17]. One of the main advantages of this technique is the ability to restore correct soft tissue tension by adjusting varus-valgus and tibial slope based on intraoperatively acquired information before performing the tibia cut [18,30]. Some authors have demonstrated that high tibial compartment forces result when the tibial component is placed more than 1° outside of the varus/valgus relative to the pre-arthritic joint line [18,30]. This technique becomes particularly useful considering the challenge of understanding pre-arthritic tibial coronal alignment and adjusting the slope, especially when the posterior cruciate ligament is sacrificed [5,6,10,12,13,14,15,31,32,33,34].

## 4. Conclusions

This paper presents an innovative approach to TKA that integrates KA principles with the NextAR AR system. KA aims to replicate the natural, pre-arthritic joint line by tailoring the prosthetic alignment to the patient’s unique knee anatomy, diverging from traditional methods that standardize alignment to a neutral axis. This individualized technique often yields more natural knee kinematics post-surgery. While resurfacing the femur generally allows for a predictable restoration of its joint line due to the consistent nature of femoral wear, restoring the tibial joint line is more complex. Tibial wear patterns are highly variable, making precise restoration challenging. To address this, CAS tools, such as AR systems, could improve the precision of tibial alignment and joint line restoration. NextAR utilizes AR to provide real-time visual guidance, overlaying critical surgical information onto the operative field. This system allows for accurate intraoperative evaluation of ligament elongation and adjustment of the cutting guides and bone thicknesses.

By combining KA and AR, this technique strives to achieve a balance between soft tissue tension and bone alignment, aiming to restore the knee’s natural function and stability. This approach is particularly beneficial in addressing the complexities of tibial alignment, offering a more accurate and individualized method for TKA. To establish the true efficacy and long-term benefits of integrating KA and AR technologies like the NextAR, further studies involving larger patient populations and comprehensive clinical data need to be performed.

## Data Availability

Data are available on request from the authors.
